# Retrospective Analysis of the SARS-CoV-2 Infection Profile in COVID-19 Positive Patients in Vitoria da Conquista, Northeast Brazil

**DOI:** 10.3390/v14112424

**Published:** 2022-10-31

**Authors:** Anna Carolina S. Dantas, Hellen B. M. Oliveira, Camila P. Gomes, Daniele L. Alves, Priscilla D. B. Infante, Rosimara de J. A. Caitité, Hegger M. Fritsch, Marina S. Cucco, Lucas S. C. Silva, Caline N. T. Oliveira, Rafaela de S. Bittencourt, Aline T. Amorim, Ana Luisa P. Nascimento, Francely A. G. C. Marinho, Danielle S. de Medeiros, Márcio G. G. de Oliveira, Sostenes Mistro, Fabricio F. de Melo, Taiana T. S. Pereira, Ana M. S. Guimarães, Jorge Timenetsky, Pablo Maciel B. Moreira, Sandra Helena P. de Oliveira, Luiz C. J. Alcantara, Marta Giovanetti, Luciane A. Santos, Vagner Fonseca, Fernanda K. Barreto, Guilherme B. Campos, Lucas M. Marques

**Affiliations:** 1 Institute of Multidisciplinary Health, Federal University of Bahia, Rua Hormindo Barros, 58, Candeias, Vitória da Conquista 45029-094, BA, Brazil; 2 Municipal Central Laboratory, Vitória da Conquista City Hall, Avenida Macaúbas, 100, Kadija, Vitória da Conquista 45065-060, BA, Brazil; 3 State University of Santa Cruz, Rod. Jorge Amado, Km 6, Salobrinho, Ilhéus 55662-900, BA, Brazil; 4 Oswaldo Cruz Institute—Fiocruz, Avenida Brasil, 4365, Manguinhos, Rio de Janeiro 21040-900, RJ, Brazil; 5 Medical School, Federal University of Bahia, Largo Terreiro de Jesus, s/n, Pelourinho, Salvador 40026-010, BA, Brazil; 6 Northeast Independent College, Avenida Luís Eduardo Magalhães, 1305, Candeias, Vitória da Conquista 45055-030, BA, Brazil; 7 Institute of Biomedical Sciences, University of São Paulo, Avenida Professor Lineu Prestes, 2415, Butantã, São Paulo 05508-900, SP, Brazil; 8 Vitória da Conquista City Hall, Rua Rotary Club, 69-Centro, Vitória da Conquista 45040-150, BA, Brazil; 9 Campus Universitário, Júlio de Mesquita Filho State University of São Paulo, Rodovia Marechal Rondon km 527/528, Araçatuba 16015-050, SP, Brazil; 10 University of Campus Bio-Medico di Roma, 00128 Rome, Italy; 11 Bahia School of Medicine and Public Heath, Av. Dom João VI, 275, Brotas, Salvador 40290-000, BA, Brazil; 12 Pan American Health Organization, Lote 19-Avenida das Nações, SEN-Asa Norte, Brasilia 70312-970, GO, Brazil

**Keywords:** SARS-CoV-2, gene expressions, sequencing, genomic and epidemiological surveillance

## Abstract

Severe Acute Respiratory Syndrome Coronavirus-2 (SARS-CoV-2) is responsible for causing Coronavirus Disease-2019 (COVID-19), a heterogeneous clinical condition that manifests varying symptom severity according to the demographic profile of the studied population. While many studies have focused on the spread of COVID-19 in large urban centers in Brazil, few have evaluated medium or small cities in the Northeast region. The aims of this study were: (i) to identify risk factors for mortality from SARS-CoV-2 infection, (ii) to evaluate the gene expression patterns of key immune response pathways using nasopharyngeal swabs of COVID-19 patients, and (iii) to identify the circulating SARS-CoV-2 variants in the residents of a medium-sized city in Northeast Brazil. A total of 783 patients infected with SARS-CoV-2 between May 2020 and August 2021 were included in this study. Clinical-epidemiological data from patients who died and those who survived were compared. Patients were also retrospectively divided into three groups based on disease severity: asymptomatic, mild, and moderate/severe. Samples were added to a qPCR array for analyses of 84 genes involved with immune response pathways and sequenced using the Oxford Nanopore MinION technology. Having pre-existing comorbidity; being male; having cardiovascular disease, diabetes, and/or chronic obstructive pulmonary disease; and PCR cycle threshold (Ct) values under 22 were identified as risk factors for mortality. Analysis of the expression profiles of inflammatory pathway genes showed that the greater the infection severity, the greater the activation of inflammatory pathways, triggering the cytokine storm and downregulating anti-inflammatory pathways. Viral genome analysis revealed the circulation of multiple lineages, such as B.1, B.1.1.28, Alpha, and Gamma, suggesting that multiple introduction events had occurred over time. This study’s findings help identify the specific strains and increase our understanding of the true state of local health. In addition, our data demonstrate that epidemiological and genomic surveillance together can help formulate public health strategies to guide governmental actions.

## 1. Introduction

The year 2020 was marked by a public health emergency of international concern, given the highest alert level of the World Health Organization (WHO). Coronavirus Disease-2019 (COVID-19), caused by Severe Acute Respiratory Syndrome Coronavirus-2 (SARS-CoV-2), was declared a pandemic on 11 March 2020 and required collective efforts from different countries and institutions to prevent the virus from spreading further [1]. During the COVID-19 pandemic, approximately 546,357,444 people were infected worldwide, with 6,336,415 deaths, according to WHO data as of 2 August 2022 [2]. Brazil, the country with the third largest number of cases worldwide, reported 33,833,900 confirmed cases and 678,514 deaths during the same period [3,4]. According to data from the epidemiological bulletin of the State Health Department, the state of Bahia reported a total of 1,662,253 confirmed COVID-19 cases and 30,377 deaths [5]. Up to 3 August 2022, Vitória da Conquista (a city in the southwest region of Bahia) registered 49,656 cases and 703 deaths, becoming the city with the third largest number of confirmed cases in Bahia [6].

The clinical symptoms of COVID-19 include fever, cough, dyspnea, myalgia, fatigue, headache, normal or decreased leukocyte count, and radiographic evidence of pneumonia [7,8]. The mean incubation period for SARS-CoV-2 is five days, and symptoms can persist for six to 41 days, with a mean of 14 days. Symptom duration and intensity are influenced by the patient’s age and immune status. Patients with pre-existing conditions, such as diabetes and respiratory and cardiovascular disorders, are more susceptible to COVID-19 [9].

Interestingly, most COVID-19 patients develop only mild symptoms or are asymptomatic during the entire period of SARS-CoV-2 infection, while others present severe symptoms that may lead to death. The reasons for these diverse manifestations are still unclear, but it has been shown and/or suggested that certain host genetic factors, as well as viral genetic variations, may influence the clinical course and outcome of COVID-19 [10,11,12,13]. In this context, understanding the disease and immune response profiles of COVID-19 patients appears to be crucial for developing effective control measures both regionally and across the country. Furthermore, clinical-epidemiological data of the affected population can complement the traditional viral genomic surveillance approach [14], providing a better resolution of disease dynamics in different regions, countries, and populations to improve the pandemic response.

According to the Brazilian Institute of Geography and Statistics (IBGE), Vitória da Conquista has an estimated population of 341,128 inhabitants and is the third largest city in the state of Bahia, Northeast Brazil. Vitória da Conquista is considered the administrative center of the entire southwest region of the state, providing a wide variety of services, especially health services, to more than 80 surrounding cities. Therefore, this city is an appropriate choice for the purposes of this study. The lack of data from this population worsens the crisis caused by the pandemic in that region because surveillance measures must be established based on data that reflect the local reality. In this context, the aims of this study were three-fold: (i) to identify risk factors for mortality from SARS-CoV-2 infection, (ii) evaluate the gene expression patterns of key immune response pathways using nasopharyngeal swabs of COVID-19 patients, and (iii) identify the circulating SARS-CoV-2 variants in the population of Vitória da Conquista, Bahia, Brazil.

## 2. Materials and Methods

### 2.1. Ethics and Patients

This study was designed in accordance with the precepts of Resolution 466/12 of the National Health Council. The procedures were initiated only after being approved by the Human Research Ethics Committee (CEPSH) of the Federal University of Bahia, under protocol no. 38544620.9.0000.5556 and with the written consent of the patients. In this study, patients were recruited after their nasopharyngeal swab samples were detected as positive for SARS-CoV-2 by the main public laboratory (Central Laboratory, LACEN) of Vitória da Conquista, Brazil. In brief, patients attended various health centers throughout the region, where their nasopharyngeal swab samples were collected and sent to LACEN for diagnostic analysis. The presence of the virus in the nasopharyngeal swab samples collected from the patients was confirmed by reverse transcriptase quantitative polymerase chain reaction (RT-qPCR), following the protocol described by the United States Centers for Disease Control and Prevention (CDC) and run in duplicate [15]. We first selected all patients with positive nasopharyngeal swabs from May 2020 to August 2021. The inclusion criteria for a patient to be included in the study were: (i) the patient’s frozen samples were in the LACEN system, (ii) the patient’s present results were recorded in the LACEN system, and (iii) the patient’s records were in the SoulMV system (a system of Vitória da Conquista City Hall). Exclusion criteria were: (i) not having a sample stored at LACEN or not having sufficient quantity to perform the tests, or (ii) no results of the patient recorded in the SoulMV system. These patients were all contacted by phone to seek their consent for research participation. Of the 1030 SARS-CoV-2 positive patients deemed eligible for inclusion, we were able to contact and acquire consent from 783 patients, who were then included in the study. Confirmed SARS-CoV-2 positive samples were aliquoted for gene expression analysis of inflammatory pathway activation markers and for viral genome sequencing.

### 2.2. Clinical-Epidemiological Data Analysis

Clinical-epidemiological data of the patients were obtained from electronic patient records in the SoulMV system. The data included age (≤19 years, 20–59 years, ≥60 years), sex (male, female), symptomatology (Table 1), residence data, the interval between symptoms and collection (≤8 days, >9 days), comorbidities (Table 1), case outcome (died or survived), disease severity (asymptomatic, mild, or moderate/severe, following the COVID-19 clinical management protocol [16]), and whether the patient was a health professional. The database was created in Microsoft Office Excel, and analyses were performed with Stata, version 15.1. A descriptive analysis of the clinical-epidemiological variables was performed by comparing absolute and relative frequencies. The association between variables was verified by Pearson’s chi-square test (Χ2), and data with a *p*-value ≤ 0.05 were considered significant. The association between variables was verified by the odds ratio (OR) at a 95% confidence interval (CI) measured using multiple logistic regression analysis. Multivariate logistic regression analysis was used for all variables that obtained a *p*-value < 0.20 in the univariate analysis.

### 2.3. Gene Expression Analysis

The gene expression patterns of selected inflammatory pathways were measured using qPCR arrays. Representative nasopharyngeal samples from patients displaying asymptomatic, mild, and moderate/severe disease and from patients who died were selected. Total RNA was extracted using the PureLink™ RNA Mini Kit (Thermo Fisher, São Paulo, Brazil) following the manufacturer’s protocol. cDNA was obtained through reverse transcription (RT) of the mRNA using the SuperScript^®^ IV Reverse Transcriptase kit (Thermo Fisher, São Paulo, Brazil) and RNAse inhibitor. cDNA was analyzed using the Human Toll-Like Receptor Signaling Pathway (Qiagen-SABioscience, São Paulo, Brazil). All procedures were performed according to the manufacturer’s instructions. For gene expression analysis, data were generated and analyzed using the kit software, and GraphPad was used to graphically represent the results. Statistical differences were considered significant at *p* < 0.05 using a 95% CI.

### 2.4. Viral Sequencing

#### 2.4.1. cDNA Synthesis and Whole Genome Sequencing

Samples were selected for sequencing based on the qPCR cycle threshold (Ct) value (≤30). SARS-CoV-2 genomic libraries were prepared using the Oxford Nanopore MinION technology. The SuperScript IV Reverse Transcriptase kit (Thermo Fisher Scientific, San Jose, CA, USA) was initially used for cDNA synthesis following the manufacturer’s instructions. The cDNA generated was subjected to multiplex PCR sequencing using Q5 High Fidelity Hot-Start DNA Polymerase (New England Biolabs, Ipswich, MA, USA) and a set of specific primers designed by the ARTIC Network for complete sequencing of the SARS-CoV-2 genome (version 3) [17]. PCR conditions were previously reported [17]. All experiments were performed in a biosafety level-2 cabinet. Amplicons were purified using 1 × AMPure XP Beads (Beckman Coulter, Brea, CA, USA) and quantified on a Qubit 3.0 fluorimeter (Thermo Fisher Scientific) using the Qubit™ dsDNA HS Assay Kit (Thermo Fisher Scientific). DNA library preparation was performed using the Ligation Sequencing Kit LSK109 (Oxford Nanopore Technologies, Oxford, UK) and the Native Barcoding Kit (NBD104 and NBD114, Oxford Nanopore Technologies, Oxford, UK). Sequencing libraries were loaded into an R9.4 flow cell (Oxford Nanopore Technologies, Oxford, UK). In each sequencing run, negative controls were used to monitor potential contamination with less than 2% mean coverage.

#### 2.4.2. Generation of Consensus Sequences

Oxford Nanopore sequencing raw files were basecalled using Guppy v3.4.5, and barcode demultiplexing was performed using qcat. Sequences were generated by de novo assembly using Genome Detective [18]. This uses DIAMOND to identify and classify candidate viral reads in broad taxonomic units using the viral subset of the Swissprot UniRef protein database. Candidate reads were next assigned to candidate reference sequences using NCBI blastn and aligned using Annotated Genome Aligner (AGA) and MAFFT. Final contigs and consensus sequences were then made available as FASTA files.

#### 2.4.3. Data Quality Control and Global Data Set Collection

In order to ensure the quality of the genome sequences generated in this study and to guarantee the highest possible phylogenetic accuracy, only genomes > 29,000 bp and <1% of ambiguities were considered (n = 71). Multiple sequence alignment was then performed with MAFFT [19], and a preliminary phylogenetic tree was inferred using the maximum likelihood in IQ-TREE2, employing the GTR + I nucleotide substitution model. Prior to further phylogenetic analysis, our data set was also assessed for both sequences with low data quality (e.g., with assembling issues, sequencing and alignment errors, data annotation errors, or sample contamination) and for molecular clock signal (i.e., temporal structure) using TempEst v1.5.3 [20]. We appended the 71 genome sequences newly generated in this project with an extensive reference data set of SARS-CoV-2 sequences, sampled and collected globally since the start of the outbreak, including near-complete genomes (n = 13,328) from Brazil (sampled up to 31 July 2021). A unique set of external references (n = 7992) were obtained by including all the non-Brazilian sequences from the global and South American NextStrain builds (date of access: 31 July 2021).

#### 2.4.4. Phylogenetic Analysis

Sequences were aligned using MAFFT [19] and submitted to IQ-TREE2 for maximum likelihood (ML) phylogenetic analysis [21] employing the general time reversible (GTR) model of nucleotide substitution and a proportion of invariable sites (+I) as selected by the ModelFinder application. Branch support was assessed using the approximate likelihood-ratio test based on the bootstrap and the Shimodaira–Hasegawa-like procedure (SH-aLRT) with 1000 replicates. The raw ML tree topology was then used to estimate the number of viral transmission events between various Brazilian regions and the rest of the world. TreeTime [22] was used to transform this ML tree topology into a dated tree using a constant mean rate of 8.0 × 10^−4^ nucleotide substitutions per site per year after the exclusion of outlier sequences.

#### 2.4.5. Lineage Classification

We used the dynamic lineage classification, as specified in the Phylogenetic Assignment of Named Global Outbreak LINeages (version 3.1.7) (PANGOLIN) protocol [23]. This aimed to identify the most epidemiologically important lineages of SARS-CoV-2 circulating in Vitória da Conquista. Both VOCs were designated based on the WHO framework as of July 2021.

## 3. Results

### 3.1. Clinical-Epidemiological Data Analysis

This study included 783 patients, most of whom were women (62.7%), aged between 20 and 59 years (62.6%), not healthcare professionals (75.2%), categorized as having moderate/severe disease (78.7%), an interval between symptoms and sample collection of up to eight days (83.1%), no comorbidity (61.7%), and Ct ≥ 23 (72.9%) (Table 2).

In the univariate logistic regression, variables significantly associated with death from COVID-19 were sex (*p* = 0.037), presence of one (*p* = 0.003) or more comorbidities (*p* = 0.001), cardiovascular disease (*p* < 0.001), diabetes (*p* = 0.002), chronic obstructive pulmonary disease (COPD) (*p* = 0.001), and Ct lower than 22 (*p* < 0.001) (Table 3). In the multivariate logistic regression, the presence of one (*p* = 0.049) or more comorbidities (*p* = 0.032) was identified as risk factors for death from COVID-19 in the studied population of Vitória da Conquista (Table 3).

### 3.2. Analysis of the Expression Profile of Inflammation Pathway Activation Markers

**Figure 1:**
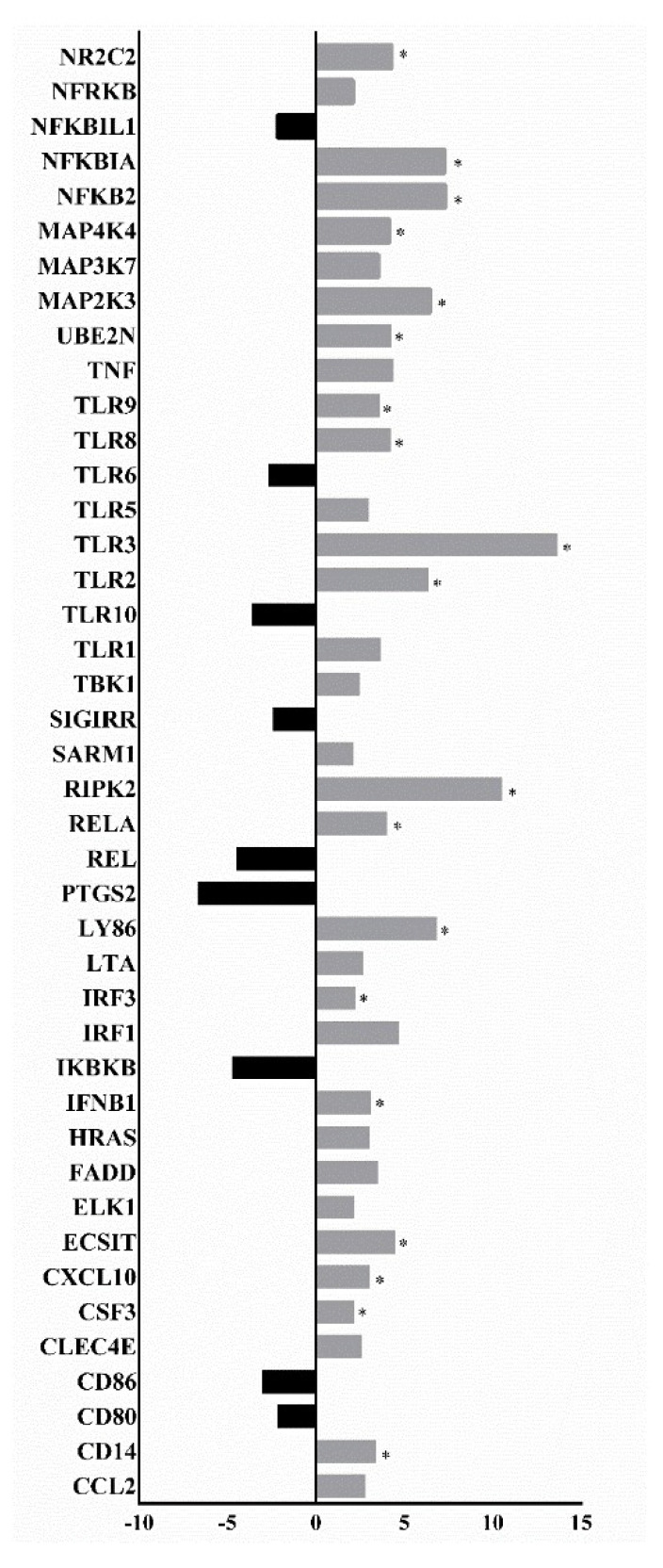
Gene expression profile comparisons between COVID-19 patients who died vs. the asymptomatic, mildly symptomatic, and moderately/severely symptomatic groups. Upregulation (gray) and downregulation (black) of genes linked to immune response activation pathways are indicated. Statistical significance (*p* < 0.05) is represented by an asterisk (*) (nonparametric Mann–Whitney One-tailed test, GraphPad Prism^®^ version 6.01).

The expression profile analysis of markers showed that different inflammatory pathways are activated after SARS-CoV-2 infection, such as the interferon (IFN), tumor necrosis factor-alpha (TNF-α), Janus kinase/signal transducers and activator of transcription (JAK/STAT), and toll-like receptor (TLR) signaling pathways. It was also inferred that NF-κB was the central signaling pathway for the infection-induced cytokine/chemokine response caused by SARS-CoV-2. Analyses of the asymptomatic, mild, and moderate/severe patients that survived compared with the group of patients who died showed statistically significant upregulation of NR2C2, NFKBIA, NFKB2, MAP4K4, MAP2K3, UBE2N, TLR9, TLR8, TLR3, TLR2, RIPK2, RELA, LY86, IRF3, IFNB1, ECSIT, CXCL10, CSF3, and CD14 expression levels (Figure 1). MYD88-dependent or independent TLR8 and TLR2 upregulation triggered increased expression of pro-inflammatory chemokines (such as CXCL10) and cytokines (such as IFNB1) in the group of patients who died. The presence of inflammatory response regulatory molecules, such as NFKBIA, negatively regulated the immune response to control the inflammatory process. Comparisons of the asymptomatic group with the patients who died showed statistically significant downregulation of TNF, TIRAP, TICAM1, NFKBIL1, LY96, JUN, CXCL8, IL6, IFNA1, HSPD1, FOS, CSF3, and CD80 expression levels (Figure S1). Comparing the asymptomatic and mildly symptomatic groups, we observed downregulation of UBE2N, TNFRSF1A, TLR8, TLR2, TLR1, TIRAP, TICAM2, RIPK2, PRKRA, MAP3K7, LY96, JUN, IL6, HSPD1, FOS, CD80, and BTK expression levels (Figure S2). Therefore, the immune response was differentially regulated in individuals showing varying levels of COVID-19 symptoms. Despite the observed downregulation in the asymptomatic group, we noted a regulated and efficient response to modulate the infection process, preventing a symptomatic condition. However, the downregulation of crucial molecules involved in the activation of the adaptive immune system can lead to the non-development of immunological memory.

The comparison between the mildly symptomatic group and the patients who died showed statistically significant differences with downregulation of TLR4 and MAPK8 and upregulation of TRAF6, TNF, TLR8, TLR5, TLR1, TIRAP, TICAM2, RIPK2, IRAK2, IL1A, IL10, IFNB1, HSPA1A, HRAS, and FADD (Figure S3). The comparison between the moderately/severely symptomatic and asymptomatic groups showed downregulation of PELI1 and upregulation of TRAF6, TOLLIP, TLR8, TLR2, TLR10, TLR1, TBK1, TAB1, RELA, REL, MAP3K1, IRF3, IRAK4, HSPA1A, CSF2, CASP8, and BTK in the moderate/severe group (Figure S4). MYD88-dependent or independent TLR8 and TLR2 upregulation and TLR10 upregulation triggered increased expression of pro-inflammatory cytokines in the moderate/severe group, such as IL1A, IL1B, IL2, IL6, IL12A, and TNF. Furthermore, we observed significantly increased expression levels of molecules involved in the cellular apoptosis pathway, including CASP8. PELI1 downregulation may act as a pro-inflammatory response regulation or compensation mechanism.

The comparison between the moderately/severely and mildly symptomatic groups showed downregulation of TOLLIP expression and upregulation of TRAF6, TLR8, TLR5, TLR2, TLR1, TAB1, MAP3K1, IRF3, IRAK4, HSPA1A, IL10, FOS, and BTK (Figure S5). Thus, MYD88-dependent or independent TLR8 upregulation can trigger increased expression of pro-inflammatory cytokines, such as TNF, IL6, IL12A, IL2, and IL1A, in the moderately/severely symptomatic group. The TLR2/TLR1 dimer can hyperactivate the expression of pro-inflammatory cytokines and increase levels of molecules related to cellular apoptosis pathways. Downregulation of TOLLIP expression reinforces the observed activation of pro-inflammatory pathways. Thus, we can infer that the greater the infection severity, the greater the activation of inflammatory pathways, triggering the cytokine storm. However, there is also the downregulation of anti-inflammatory pathways. In addition, upregulation of molecules related to cellular apoptosis pathways, the NF-κB pathway, cell death, and tissue injury caused by the infection was observed, and there was activation of antiviral response pathways marked by the production of cytokines such as CXCL10 and IFN type I.

### 3.3. SARS-CoV-2 Genomic Data

**Figure 2:**
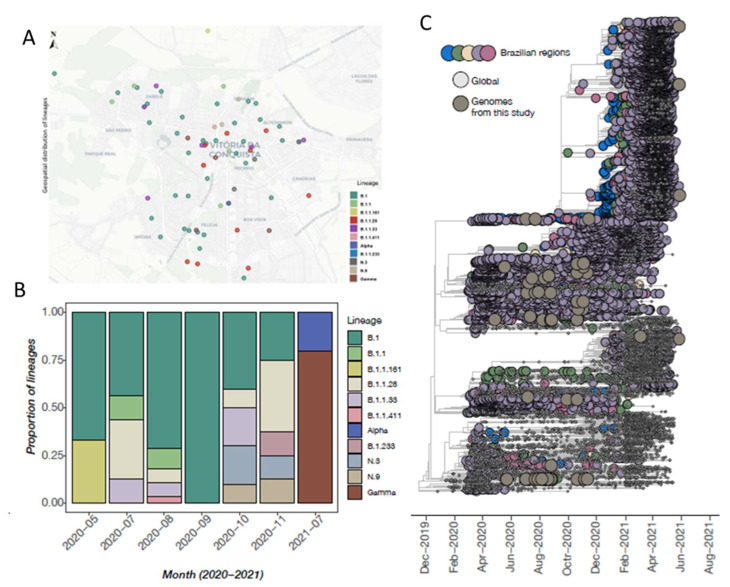
SARS-CoV-2 sequencing in Vitória da Conquista, BA, Brazil. (**A**) Distribution of sequenced samples among the 34 municipality districts; (**B**) Lineages found in the municipality between May 2020 and August 2021; (**C**) Phylogenetic analysis and lineage diversity between December 2019 and August 2021.

A total of 71 nearly complete genome sequences of SARS-CoV-2 were obtained from RT-qPCR-positive samples as part of this study. SARS-CoV-2 sequencing spanned from May 2020 to August 2021, and samples were collected from 34 distinct regions/units of the city (Table S1 and Figure 2A). The sequenced samples were collected from 29 women and 42 men (Table S1) with a median age of 51 years (range: 0 to 88 years of age). All tested samples contained sufficient viral genetic material (≥2 ng/µL) for library preparation. For positive samples, PCR Ct values were, on average, 21.77 (range: 9.0 to 25.97). Sequences had a median genome coverage of 93% (range: 80.0 to 99.8). Epidemiological information and sequencing statistics of the generated sequences from Brazil are detailed in Table S1. Sequences were assigned to nine different PANGO lineages based on the proposed dynamic nomenclature for SARS-CoV-2 lineages (Figure 2B and Table S1) and have been submitted to GISAID following the WHO guidelines (version 3.1.7, August 2021).

### 3.4. Phylogenetic Inference and Lineage Diversity

The time-stamped phylogeny deduced in this study revealed that the newly obtained sequences are scattered throughout the tree, highlighting that multiple independent introduction events have occurred over time (Figure 2A). In addition, the sequenced strains clustered with viral strains isolated in other Brazilian regions (mainly southeastern states), suggesting that those regions likely acted as a source in the dissemination of the virus (Figure 2A). This possibly was influenced by increased human mobility.

## 4. Discussion

The COVID-19 pandemic was a scenario that the public health community had feared for decades, with a new virus rapidly spreading worldwide [24]. The impact of this situation will still be noticed in all aspects of life. It is crucial to identify the origin and evolutionary path of a pathogen causing a pandemic to implement adequate control measures and help prevent future pandemics [25]. The spectrum of the disease intensity caused by SARS-CoV-2 infection varies widely, from asymptomatic to mild, severe, and even fatal, with the condition described as a cytokine storm being associated with COVID-19 severity [26,27]. It is important to recognize the profiles of the affected patients and understand the risk factors in a given population. In addition, recognizing up- or downregulated inflammatory or anti-inflammatory pathways according to infection symptom severity and elucidating the different circulating SARS-CoV-2 strains will improve the understanding of virus behavior in the population studied.

This study analyzed data from 783 SARS-CoV-2 positive patients, as confirmed by RT-PCR, from Vitória da Conquista, Bahia, Brazil. The samples were collected from May 2020 to August 2021. Our findings show that men with one or more comorbidities, especially cardiovascular disease, diabetes, and/or COPD, and with a PCR Ct value lower than 22 are significantly more vulnerable to succumbing to this disease. Our data corroborate a meta-analysis reporting that older men with comorbidities (especially chronic kidney disease, COPD, and coronary heart disease) presented strong epidemiological evidence of associations with COVID-19 severity and prognosis [28]. Other studies also reported a relatively higher risk of men developing the severe form of the disease, thus identifying a patient’s sex as a risk factor for hospital admission and higher vulnerability to a more severe manifestation of the disease [29,30].

Some studies show that the percentage of hospitalized men in Brazil was significantly higher when compared with women, with 55.96% of the 1,765,200 hospitalized cases reported until November 2021 being men. In addition, of the 562,851 patients who died in the same month, 56.19% were men [31]. Other authors highlighted that most patients who died from COVID-19 were men, with values ranging from 59% to 75% of total mortality. Strong evidence suggests that sexual dimorphism plays a central role in the genetic and hormonal regulation of both the innate and adaptive immune system responses, especially in the context of viral infections [32].

The analyses of comorbidities, especially cardiovascular diseases, diabetes, and COPD, showed that their presence could worsen the infection prognosis, as they can cause lung injury and often death. A retrospective observational study in patients with hypertension reported a two-fold increase in COVID-19 mortality rates, highlighting a direct relationship between hypertension and COVID-19 [33]. Diabetes was associated with greater weight loss and increased inflammation in COVID-19 patients [34]. Other authors showed that the number of comorbidities is a significant predictor of mortality [35]. Compared with single present comorbidities, the presence of more than one entailed a greater risk of death. A study reported that SARS-CoV-2 infection becomes even more harmful in patients with comorbidity and that the appropriate clinical management of these patients is a crucial step for their survival [36]. Other authors reported that comorbidities are potential risk factors for COVID-19 patients in the Intensive Care Unit (ICU) [37]. Patients with heart, lung, and/or metabolic diseases are more prone to SARS-CoV-2 infection because of a dysregulated immune response. Thus, the data from our study can provide subsidies for the individualized implementation of SARS-CoV-2 infection treatment and prevention, in addition to data that ratify new treatment strategies alone or combined with other therapies for SARS-CoV-2 infection, which result in more precise interventions.

The main technique recommended for the diagnosis of SARS-CoV-2 infection is RT-qPCR, as it provides the sample Ct. Some studies investigated the relationships between Ct values, epidemiological data, and infection severity and presented controversial results, with its clinical relevance still being discussed and its use still not implemented in clinical practice [38,39,40]. Here, we analyzed different Ct values according to COVID-19 severity. Significantly different results were observed between groups, with higher Ct values in the asymptomatic group and lower Ct values in severe cases and in cases leading to death. A study that correlated Ct with epidemiological data demonstrated lower Ct values at the onset of SARS-CoV-2 infection symptoms and in patients reporting respiratory symptoms at the time of collection [38]. Another cohort study associated lower Ct with a higher risk of death [41]. One report described increased viral load in samples of respiratory origin in patients with the more aggressive disease compared with those with more moderate courses [42]. Consequently, the Ct value could be used as a possible prognostic indicator and could be included in RT-qPCR reports issued by the laboratories, thus helping in decision making about infection control. We also emphasize that the Ct should be analyzed by health managers because of its apparent importance.

In general, our study showed that the greater the severity of the infectious condition caused by SARS-CoV-2, the greater the expression of pro-inflammatory cytokines and chemokines, such as IL-6, IFN, and TNF. This upregulation triggers an uncontrolled inflammatory response that plays a key role in COVID-19 pathogenesis. In addition, increased expression levels of molecules involved in the cellular apoptosis pathway were observed, including upregulation of MAP3K7 and FADD. The pathogenesis of SARS has not been fully elucidated. Some analyses showed that the development of this condition involves an imbalance between pro- and anti-inflammatory mechanisms and the interaction of several cell types and cytokines, resulting in disordered immune regulation [33]. A study by Jose et al. published in 2020 associated the outcome of death in patients with severe COVID-19 with high levels of circulating pro-inflammatory cytokines [43], which was also observed in our study with TNF and IL1 expression. The comparison between the patients who died and the asymptomatic, mildly symptomatic, and moderately/severely symptomatic groups showed IFNB1 overexpression. IFN is associated with more powerful innate immune responses in preventing viral replication in the initial phase of infection and with the quick elimination of viruses from infected cells [44]. Other authors showed that inborn errors of IFN signaling and the presence of anti-IFN autoantibodies could worsen the condition of COVID-19 patients, demonstrating the relevance of IFN immunity in this infection [44,45].

The inflammatory cytokines TNF-α and IFN-γ play critical roles in COVID-19 pathogenesis through their induction of cell death [46]. Inhibition of the TNF-α-NF-κB inflammatory pathway in COVID-19 patients may prevent pulmonary complications [47]. The JAK/STAT1 pathway controls IRF1 transcription regulation, which is critical for the death of inflammatory cells in response to TNF-α and IFN [46]. SARS-CoV-2 disrupts the JAK/STAT pathway, limiting IFN signaling and facilitating viral replication. Data from another study showed that the SARS-CoV-2 acts on JAK, tyrosine kinase 2 (Tyk2), and interferon receptor (IFNAR1), resulting in cellular desensitization to type I IFN [48]. These molecules were also observed to be significantly expressed in our study. TLR4 plays a role in the progression of SARS-CoV-2 infection by activating the production of type I interferons and pro-inflammatory cytokines to fight the infection [49]. TLRs, mainly TLR3, TLR7, and TLR8, are present in plasmacytoid dendritic cells and are the main recognition sensor of SARS-CoV-2 [50].

In our study, we observed that the greater the severity of the infection, the greater the expression of TLR8, which is a molecule highly expressed in the lung. It can serve as a good prognostic marker. Another study showed that TLR1, TLR4, TLR5, TLR8, and TLR9 expression levels were significantly increased in severe and critical cases and that TLR7 expression was observed in patients with moderate conditions [51]. We can hypothesize that the action of the virus, the immune response, and the release of pro-inflammatory factors favor the cytokine storm in COVID-19 pathogenesis, which is directly related to infection severity.

During replication, viruses continually accumulate genomic mutations that persist due to natural selection. This concept is also true for SARS-CoV-2. These mutations improve the virus’s ability to proliferate and infect, in addition to facilitating evasion of the host’s immune response. Given this scenario, epidemiological and genomic surveillance is crucial for tracking the path of a virus to reach a specific location [52,53,54]. This strategy also reveals the impacts of viral mutations on transmissibility, infection, and the ability to evade the host’s immune response. In Brazil, the dissemination of new SARS-CoV-2 strains in different Brazilian regions and cities is critical, as new stains have emerged since the beginning of the pandemic.

This study analyzed the sequencing results of 71 samples representing different regions of the municipality between May and November 2020 and July 2021, identifying different strains with independent introduction times that may have been influenced by human mobility. The first lineages identified were B.1 and B.1.1.161. Lineage B.1 was found in all analyses of our study from May to November 2020 and distributed in all regions of the municipality. In Brazil, B.1 has been reported since February 2020 and is described for the first time in São Paulo. Lineage B.1.1.161 was reported for the first time in Minas Gerais in March 2020 [52]. This lineage was detected in our study in May of the same year, once again showing the circulation of the virus. Vitória da Conquista is considered a regional capital that has geographic proximity to the cities of the northern region of Minas Gerais, with great population mobility in this area. Lineage B.1.1.28 is potentially emerging in Brazil from the Amazon region. B.1.1.28 and B.1.1.33 began to appear in February 2020, showing spike protein mutations and dominating the observed cases in Brazil [55]. These strains were observed in samples from July 2020. Lineages N3 and N9 were identified in October 2020, with N9 derived from B.1.1.33, identified earlier in the present study.

The Gamma variant was initially detected in Japan and Brazil and was described for the first time in Brazil in the city of Manaus in December 2020. It became a variant of interest because of the mutations found mainly in the E484K, K417T, and N501Y proteins [56,57]. This variant was detected in Vitória da Conquista seven months later, in July 2021. The Gamma lineage predominated in the second wave of the pandemic in Brazil and was widespread throughout all demographic regions of the country by the end of December 2020 [58]. The Alpha variant (also called B.1.1.7) was observed in September 2020 in the UK, becoming the dominant strain in December 2020 when it was identified in Brazil [59]. This variant was detected by our study in July 2021. Thus, this study emphasizes the importance of SARS-CoV-2 genomic surveillance to monitor virus spread in different regions, directly impacting public health in the municipal and Brazilian states.

The present study has some limitations. First, the absence of healthy group controls since the gene expression profile might be different among healthy people and asymptomatic, mildly symptomatic, and moderately/severely symptomatic groups. Second, the expression levels of molecules were examined at the gene level, not the actual protein level, as well as whether different SARS-CoV-2 variants play a role in the examined gene expression profile difference. Third, this article was the result of a partnership project between the Federal University of Bahia, the municipal government, and the Municipal Central Laboratory, with the objective of providing the diagnosis of SARS-CoV-2 infection in the municipality, avoiding forwarding the diagnosis to laboratories in the state capital. Unfortunately, the municipality ended the molecular diagnosis from the moment the rapid tests appeared (due to cost), which ended our access to new samples.

## 5. Conclusions

This study demonstrates the importance of performing genomic and epidemiological surveillance to develop better public health strategies. In addition, considering pathologies that do not have their pathogenesis clarified yet, such as COVID-19, there is an evident need to analyze these data together with analyses of the inflammatory or anti-inflammatory pathways involved in the infection. Quickly identifying strains can help reduce transmission in the community because mutations can cause relevant clinical-epidemiological changes with different severities and greater potential for infectivity.

## Figures and Tables

**Table 1:** Symptoms and comorbidities of COVID-19 patients in Vitória da Conquista, BA, Brazil.

Symptoms	Comorbidities
Fever Low fever or chills Cough Odynophagia	Cardiovascular disease Hypertension Diabetes Liver disease
Coryza Nasal congestion Mild breathing difficulty Progressive breathing difficulty Ageusia or anosmia Urinary deficit Syncope SpO_2_ saturation lower than 94% Mental confusion Signs of cyanosis Drowsiness Fast or slow breathing Myalgia/arthralgia Diarrhea Emesis Headache Irritability/confusion Adynamia Sputum production Conjunctival congestion Dysphagia Lymphadenopathy Nasal flaring Dehydration Inappetence	Chronic neurological or neuromuscular disease Immunosuppression Human Immunodeficiency Virus (HIV) Kidney disease Chronic lung disease Neoplasia (solid or hematological tumor) Obesity Asthma

**Table 2:** Sociodemographic and clinical characteristics of COVID-19 patients in Vitória da Conquista, BA, Brazil.

Variables	n	%
**Sex**		
Male	292	37.3
Female	491	62.7
**Age (years)**		
≤19	50	6.4
20–59	490	62.6
≥60	243	31.0
**Health professional**		
Yes	194	24.8
No	589	75.2
**Disease severity**		
Asymptomatic	36	4.6
Mildly symptomatic	131	16.7
Moderately/severely symptomatic	616	78.7
**Interval between appearance of symptoms and collection of sample**		
≤8 days	621	83.1
>9 days	126	16.9
**Number of comorbidities**		
0	483	61.7
1	205	26.2
≥2	95	12.1
**Ct ^a^**		
<22	212	27.1
≥23	571	72.9

^a^ Cycle threshold.

**Table 3:** Risk factors for death from COVID-19 in patients from Vitória da Conquista, BA, Brazil.

Variables	Disease Severity ^a^ n = 783	Death n = 35	OR (Crude)			OR (Adjusted) ^b^		
			OR	CI 95%	*p*-Value	OR	CI 95%	*p*-Value
	n (%)							
**Age (years)**								
≤19	50 (6.39)	1 (2.85)						
20–59	490 (62.58)	7 (20.0)	0.74	0.09–5.89	0.751	0.47	0.06–4.03	0.493
≥60	243 (31.03)	27 (77.15)	6.12	0.81–46.17	0.079	3.18	0.40–25.18	0.274
**Sex**								
Male	292 (37.29)	19 (54.3)	**0.48**	**0.24–0.95**	**0.037** *	0.53	0.26–1.07	0.078
Female	491 (62.71)	16 (45.7)						
**Number of comorbidities**								
0	483 (61.7)	11 (31.4)						
1	205 (26.2)	15 (42.9)	**3.39**	**1.53–7.51**	**0.003** *	**2.33**	**1.00–5.41**	**0.049** *
≥2	95 (12.1)	9 (25.7)	**4.49**	**1.81–11.16**	**0.001** *	**2.88**	**1.10–7.56**	**0.032** *
**Cardiovascular disease**								
Yes	645 (82.38)	16 (45.7)	**4.32**	**2.16–8.63**	**<0.001** *			
No	138 (17.62)	19 (54.3)						
**Diabetes**								
Yes	678 (86.59)	11 (31.4)	**3.19**	**1.51–6.72**	**0.002** *			
No	105 (13.41)	24 (68.6)						
**Obesity**								
Yes	713 (91.06)	2 (5.7)	**0.61**	**0.14–2.58**	**0.498**			
No	70 (8.94)	33 (94.3)						
**COPD** ^c^								
Yes	740 (94.51)	32 (91.4)	**11.59**	**2.77–48.46**	**0.001** *			
No	43 (5.49)	3 (8.6)						
**Ct** ^d^								
<22	212 (27.1)	21 (60.0)	**3.41**	**1.72–6.77**	**<0.001** *	**3.36**	**1.64–6.90**	**0.001** *
≥23	571 (72.9)	14 (40.0)						
**Interval between symptoms and sample collection**								
≤8 days	621 (83.1)	30 (85.7)						
>9 days	126 (16.9)	5 (14.3)	0.81	0.31–2.14	0.677			

^a^ Disease severity: asymptomatic, mildly symptomatic, and moderately/severely symptomatic; ^b^ Adjusted; ^c^ COPD: chronic obstructive pulmonary disease; ^d^ Ct: Cycle threshold. Statistical significance (*p* < 0.05) is represented by an asterisk (*)

## Data Availability

Not applicable.

## Supplementary Materials

The following supporting information can be downloaded at: https://www.mdpi.com/article/10.3390/v14112424/s1, Figure S1: Gene expression profile comparison between the asymptomatic group vs. patients who died. Upregulation (gray) and downregulation (black) of genes linked to immune response activation pathways are indicated. Statistical significance (*p* < 0.05) is represented by an asterisk (*) (nonparametric Mann–Whitney One-tailed test, GraphPad Prism^®^ version 6.01). Figure S2: Gene expression profile comparison between the asymptomatic and mild groups. Upregulation (gray) and downregulation (black) of genes linked to immune response activation pathways are indicated. Statistical significance (*p* < 0.05) is represented by an asterisk (*) (nonparametric Mann–Whitney One-tailed test, GraphPad Prism^®^ version 6.01). Figure S3: Gene expression profile comparison between the mild group vs. patients who died. Upregulation (gray) and downregulation (black) of genes linked to immune response activation pathways are indicated. Statistical significance (*p* < 0.05) is represented by an asterisk (*) (nonparametric Mann–Whitney One-tailed test, GraphPad Prism^®^ version 6.01). Figure S4: Gene expression profile comparison between the moderate/severe and asymptomatic groups. Upregulation (gray) and downregulation (black) of genes linked to immune response activation pathways are indicated. Statistical significance (*p* < 0.05) is represented by an asterisk (*) (nonparametric Mann–Whitney One-tailed test, GraphPad Prism^®^ version 6.01). Figure S5: Gene expression profile comparison between the moderate/severe and mild groups. Upregulation (gray) and downregulation (black) of genes linked to immune response activation pathways are indicated. Statistical significance (*p* < 0.05) is represented by an asterisk (*) (nonparametric Mann–Whitney One-tailed test, GraphPad Prism^®^ version 6.01). Table S1: Characteristics of patients and sequenced samples.
