# Supplementary material for: Retrospective Analysis of the SARS-CoV-2 Infection Profile in COVID-19 Positive Patients in Vitoria da Conquista, Northeast Brazil
Source: Viruses. 2022 Oct 31;14(11):2424. doi: 10.3390/v14112424 (PMC9699198; doi:10.3390/v14112424)
Supplement: Supplementary file 1 [file viruses-14-02424-s001.zip › viruses-1972133-supplementary.pdf]

Supplementary material

Table S1. Characteristics of patients and sequenced samples

Identification	Sex	Age	Ct[a]	Disease severity	Neighborhood	Collection[b]	Reads	Coverage	Depth of Coverage	Identity NT	Identity AA	Lineages
VC5	M	44	20,81	Asymptomatic	Felícia	5/23/2020	210629	92.5	4402.5	99.9	99.7	B.1
VC7	M	81	18,15	Moderate /severe	Cruzeiro	5/22/2020	315998	96.6	6637.7	100	100	B.1
VC11	M	52	21,1	Asymptomatic	Campinhos	10/5/2020	290884	99.5	6102.7	99.9	99.9	B.1.1.33
VC12	M	55	18,4	Mild	Ibirapuera	8/19/2020	336426	99	6465.4	100	99.9	B.1
VC21	F	61	23,69	Moderate /severe	Urbis V	7/23/2020	245351	96.5	5008.7	99.8	99.9	B.1
VC34	M	50	20,22	Mild	Urbis VI	8/13/2020	265303	95.7	7779	99.9	99.9	B.1
VC38	M	48	24,05	Moderate /severe	Boa Vista	7/3/2020	142551	99.7	2701.6	99.9	99.9	B.1.1.28
VC44	F	45	18.75	Moderate /severe	Alto da Boa Vista	8/20/2020	263139	96.3	5428.4	99.9	99.9	B.1
VC46	F	47	20,99	Moderate /severe	Morada dos Pássaros I	8/25/2020	180668	92.4	5379.1	99.8	99.6	B.1
VC49	M	42	24,98	Moderate /severe	Urbis III	8/25/2020	306933	92.1	6507.3	100	99.9	B.1
VC50	M	33	21,83	Moderate /severe	Urbis V	8/26/2020	293570	94.8	10544.9	99.9	99.9	B.1
VC51	M	63	19,02	Mild	Cidade Maravilhosa	8/26/2020	198407	92.9	4128.9	99.9	99.8	B.1
VC52	M	54	18,97	Moderate /severe	Morada dos Pássaros III	8/27/2020	199091	84.6	4455.7	100	99.8	B.1
VC53	F	40	23,32	Moderate /severe	Santa Cecilia	8/27/2020	310881	94.7	6342.7	100	99.9	B.1
VC54	M	49	24,12	Moderate /severe	Kadija	8/27/2020	202273	92.1	4166.9	99.9	99.8	B.1
VC56	F	48	21,58	Moderate /severe	Zabele	8/27/2020	271181	90.5	5780.5	99.9	99.8	B.1
VC60	F	86	22,71	Asymptomatic	Patagônia	10/21/2020	246885	91	5217.9	99.9	99.8	B.1
VC61	M	86	25,5	Moderate /severe	Bairro Brasil	10/21/2020	223013	94	6439.9	99.9	99.9	B.1
VC66	M	35	19,89	Moderate /severe	Guarani	11/2/2020	173960	99.4	3305.8	99.9	99.9	N.9
VC68	F	30	21,46	Moderate /severe	Primavera	11/03/2020	176536	94	3742.1	100	99.9	B.1.233
VC81	F	50	25,6	Asymptomatic	Bairro Brasil	8/20/2020	185269	99.5	3849.3	99.9	99.9	B.1.1.33
VC90	M	65	19,08	Mild	Recreio	11/6/2020	320774	97.3	6604.9	100	99.9	B.1
VC92	M	30	23.46	Asymptomatic	Felícia	7/21/2021	198125	99.5	3855.3	99.8	99.8	P.1
VC96	M	32	25.5	Recovered	Boa Vista	7/21/2021	165078	99.4	3587.9	99.8	99.8	P.1
VC6	F	62	15.13	Moderate /severe	Nossa Senhora de Lourdes	5/18/2020	300591	98.2	5672.7	99.9	99.8	B.1.1.161
VC14	M	48	17,77	Mild	Jurema	7/2/2020	413780	98.4	8805.6	99.9	99.9	B.1
VC17	M	68	23,09	Moderate /severe	Vila Serrana II	7/3/2020	212703	97.1	5918.6	99.9	99.8	B.1
VC18	F	6	22,13	Moderate /severe	Vila Serrana III	7/5/2020	392051	99.4	6658.8	99.9	99.8	B.1.1
VC26	M	44	17,2	Death	Jardim Sudoeste	7/29/2020	360095	99.3	7679.1	99.9	99.8	B.1
VC28	M	29	16,74	Moderate /severe	Jardim Guanabara	7/30/2020	284781	99.5	5993.6	100	99.9	B.1.1.28

VC29	M	71	19,21	Moderate /severe	Santa Cecilia	8/6/2020	404282	99.7	11050.8	99.9	100	B.1
VC32	F	45	20,11	Moderate /severe	Bairro Brasil	8/08/2020	234122	99.7	4782.6	99.9	99.8	B.1
VC33	M	77	24,2	Mild	Cruzeiro	8/12/2020	263720	99.4	5170.5	99.9	99.9	B.1
VC35	M	40	21,92	Moderate /severe	Recreio	8/14/2020	308326	99.7	6703.6	99.9	99.9	B.1.1.33
VC39	M	50	22,63	Moderate /severe	Conveima I	8/14/2020	297037	99.4	6263.4	99.9	99.9	B.1
VC40	F	61	24,36	Moderate /severe	Nossa Senhora Aparecida	8/20/2020	123639	99.4	2473.8	100	100	B.1
VC42	M	75	22,45	Moderate /severe	Felícia	8/18/2020	268623	99.5	5563.9	99.9	99.9	B.1
VC45	F	42	24,77	Moderate /severe	Ayrton Sena	8/21/2020	177515	99.6	3600.6	99.9	99.9	B.1.1.28
VC58	M	15	24,23	Moderate /severe	Boa Vista	10/21/2020	141679	99.7	2935.6	99.9	99.9	N.3
VC62	M	41	20,72	Moderate /severe	Espírito Santo	10/21/2020	106549	99.4	2390	99.9	99.9	B.1.1.28
VC64	F	64	22,08	Moderate /severe	Vila Serrana I	10/21/2020	95620	99.1	2143	99.9	99.9	B.1.1.33
VC67	M	34	22,86	Moderate /severe	Primavera	11/3/2020	215105	99.5	4575.5	99.9	99.9	N.3
VC70	M	58	21,27	Moderate /severe	Alto Maron	11/4/2020	211953	99.4	4240.8	99.9	99.9	B.1.1.28
VC71	M	86	23,65	Moderate /severe	Bairro Brasil	11/15/2020	299318	99.4	5950.1	99.9	99.9	B.1.1.28
VC77	F	50	24,14	Moderate /severe	Patagônia	7/16/2020	199489	99.5	4546.8	100	99.9	B.1
VC79	M	42	20,88	Mild	Morada dos Pássaros III	7/3/2020	208639	99.4	4268.1	99.7	99.6	B.1
VC94	F	13	19.0	Recovere d	Nossa Senhora de Lourdes	7/22/2021	260402	99.5	5103.9	99.8	99.8	P.1
VC98	M	69	19.58	Mild	Centro	7/21/2021	243410	99.5	4434.4	99.8	99.8	P.1
VC1	F	74	25,68	Moderate /severe	Recreio	11/11/2020	209933	99.5	3626.7	99.9	99.9	B.1.1.28
VC4	F	52	25,13	Mild	Recreio	10/20/2020	246220	99.5	4361.3	99.9	99.9	N.3
VC8	F	49	24,34	Mild	Vila Serrana II	9/29/2020	219811	94.1	4375.9	99.8	99.6	B.1
VC9	F	43	18,97	Mild	Centro	8/5/2020	194932	91.1	3797.8	99.9	99.7	B.1
VC10	F	42	24,32	Asympto matic	Nossa Senhora Aparecida	8/26/2020	179662	95.2	3318	99.9	99.8	B.1.1
VC19	F	34	22,91	Asympto matic	Alegria	7/9/2020	279529	99.5	4906	99.9	99.9	B.1.1.28
VC20	M	27	22,2	Moderate /severe	Primavera	7/12/2020	220287	99.7	3800.1	99.9	99.9	B.1.1.33
VC22	F	34	24,92	Moderate /severe	Sumaré	7/23/2020	185269	99.7	3431.3	99.9	100	B.1.1.28
VC36	M	55	24,66	Moderate /severe	Alto Maron	8/17/2020	220143	91	4304	99.9	99.7	B.1
VC37	M	0	26,15	Asympto matic	Cidade Maravilhosa	7/7/2020	262056	99.8	4845.2	99.9	100	B.1.1.33
VC41	M	48	20,18	Moderate /severe	Miro Cairo	8/17/2020	249448	99.7	4600.7	99.9	99.9	B.1.1
VC43	M	57	21.68	Moderate /severe	Recanto dos Pássaros	8/20/2020	139124	91.3	2650.6	99.9	99.8	B.1
VC47	F	54	9,0	Moderate /severe	Candeias	7/16/2020	243308	99.5	4520.8	99.9	99.9	B.1.1.28
VC48	F	79	23,49	Moderate /severe	São Vicente	8/26/2020	29191	94.7	526.6	99.9	99.7	B.1.1
VC57	F	27	25.97	Mild	Morada dos Pássaros III	10/22/2020	193380	90.7	3694.8	99.8	99.7	B.1
VC63	M	20	19,18	Moderate /severe	Recreio	10/21/2020	166456	90	3254	99.9	99.8	B.1

VC65	M	77	23,06	Mild	Iracema	10/23/2020	234097	99.7	4026	99.9	99.9	N.9
VC72	M	62	21,69	Moderate /severe	Ibirapuera	11/12/2020	154542	80.9	3323.6	99.9	99.6	B.1
VC75	M	67	21,78	Death	Felícia	7/23/2020	133418	99.4	3311.6	100	99.9	B.1.1
VC83	F	78	22,03	Moderate /severe	Bairro Brasil	7/23/2020	161015	95.7	2936.3	99.9	99.7	B.1
VC87	F	88	22,11	Death	Ayrton Sena	8/12/2020	195353	99.5	3329.2	99.9	99.9	B.1.1.28
VC91	M	84	25.59	Death	Ibirapuera	8/25/2020	237911	99.8	4433.5	99.9	99.9	B.1.1.411
VC97	F	32	22,81	Recovere d	Boa Vista	7/21/2021	244890	99.7	4226.2	99.9	99.8	B.1.1.7

[a] Cycle threshold [b] Sample collection date

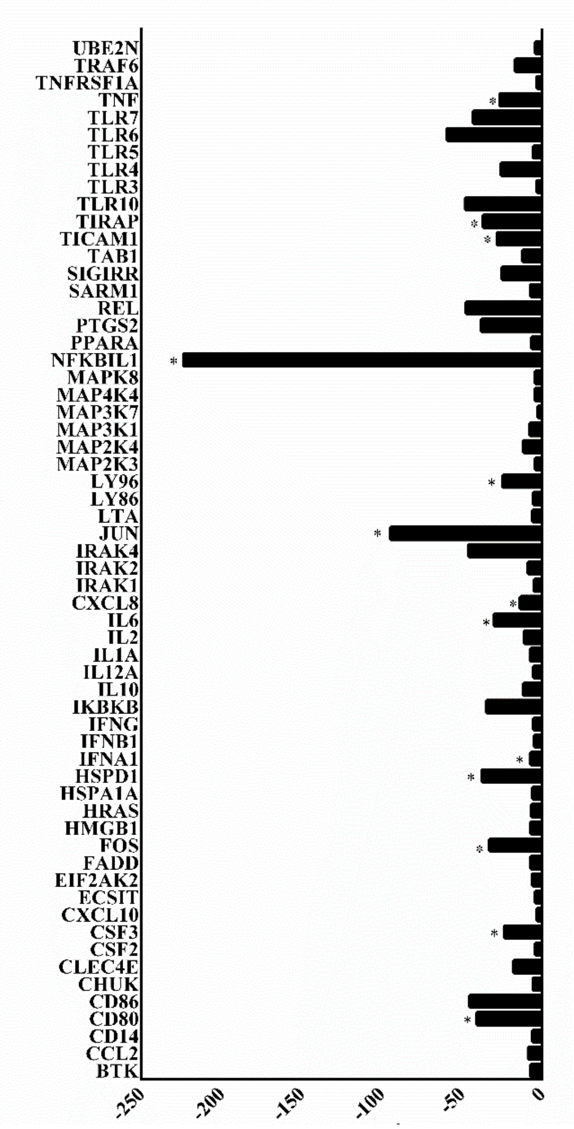

Figure S1. Gene expression between asymptomatic group vs death. Upregulation (gray) and downregulation (black) of genes linked to immune response activation pathways. Statistical significance ($p < 0.05$) represented by an asterisk (*) (nonparametric Mann-Whitney-One-tailed test, GraphPad Prism® version 6.01).

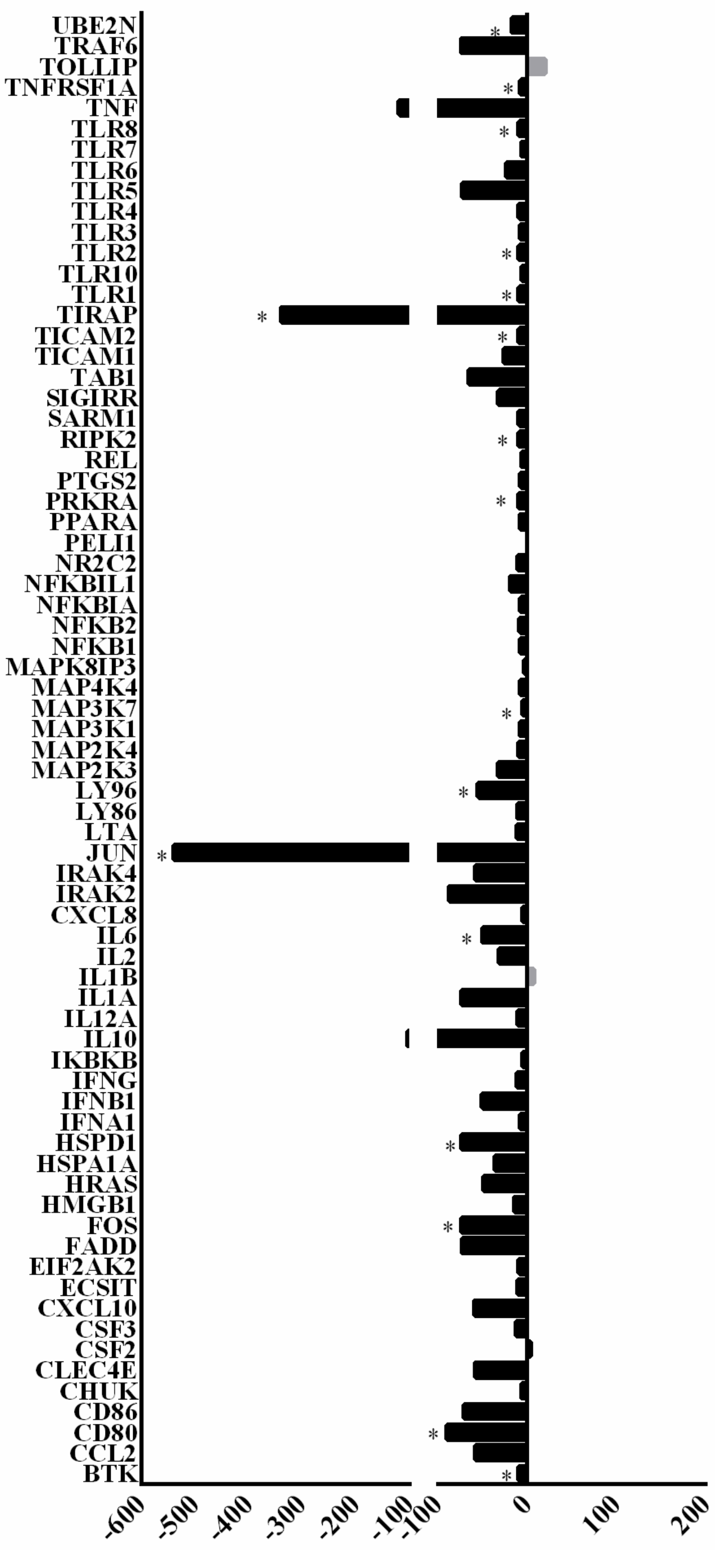

Figure S2. Gene expression between the asymptomatic vs mild groups. Upregulation (gray) and downregulation (black) of genes linked to immune response activation pathways. Statistical significance ($p < 0.05$) represented by an asterisk (*) (nonparametric Mann-Whitney-One-tailed test, GraphPad Prism® version 6.01).

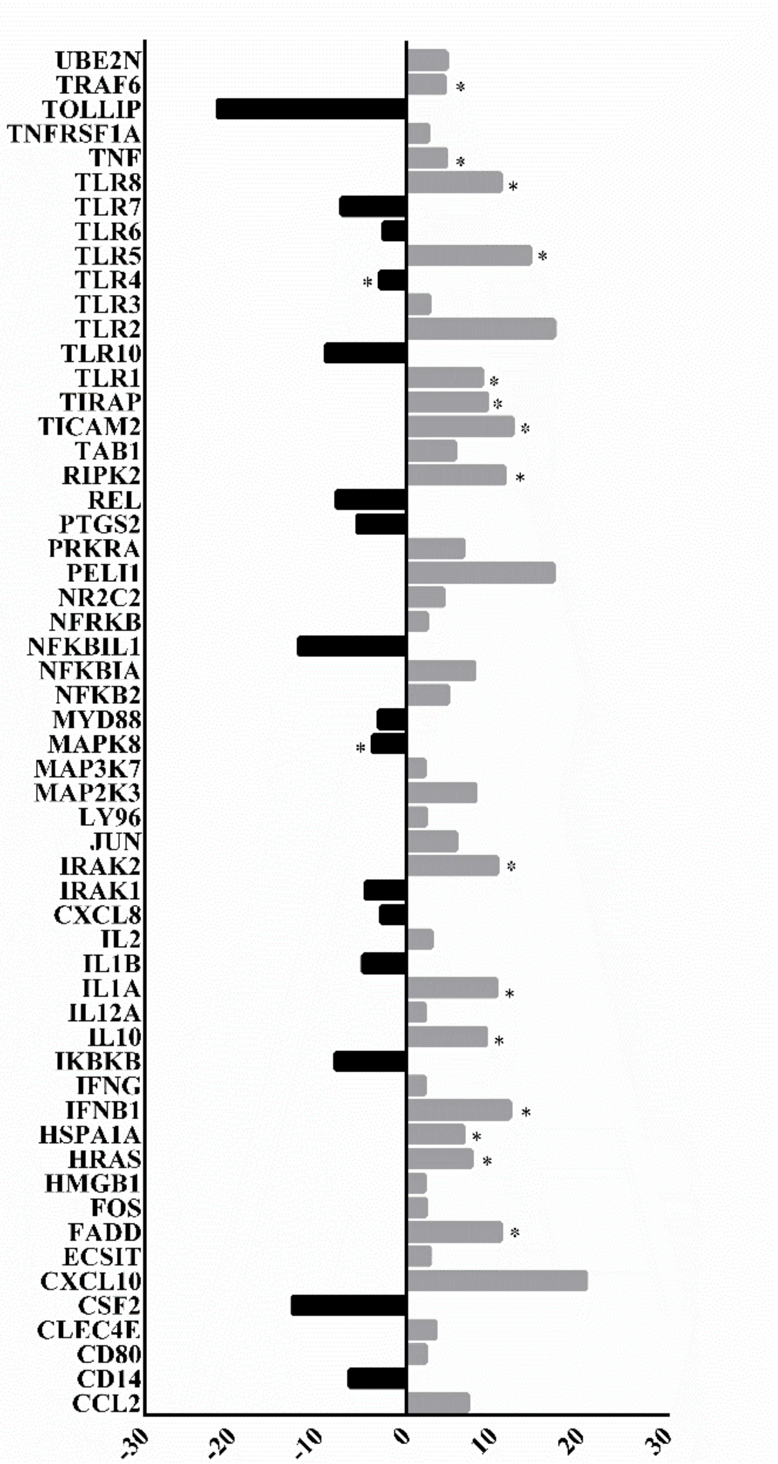

Figure S3. Gene expression between the mild group vs death. Upregulation (gray) and downregulation (black) of genes linked to immune response activation pathways. Statistical significance ($p < 0.05$) represented by an asterisk (*) (nonparametric Mann-Whitney-One-tailed test, GraphPad Prism® version 6.01).

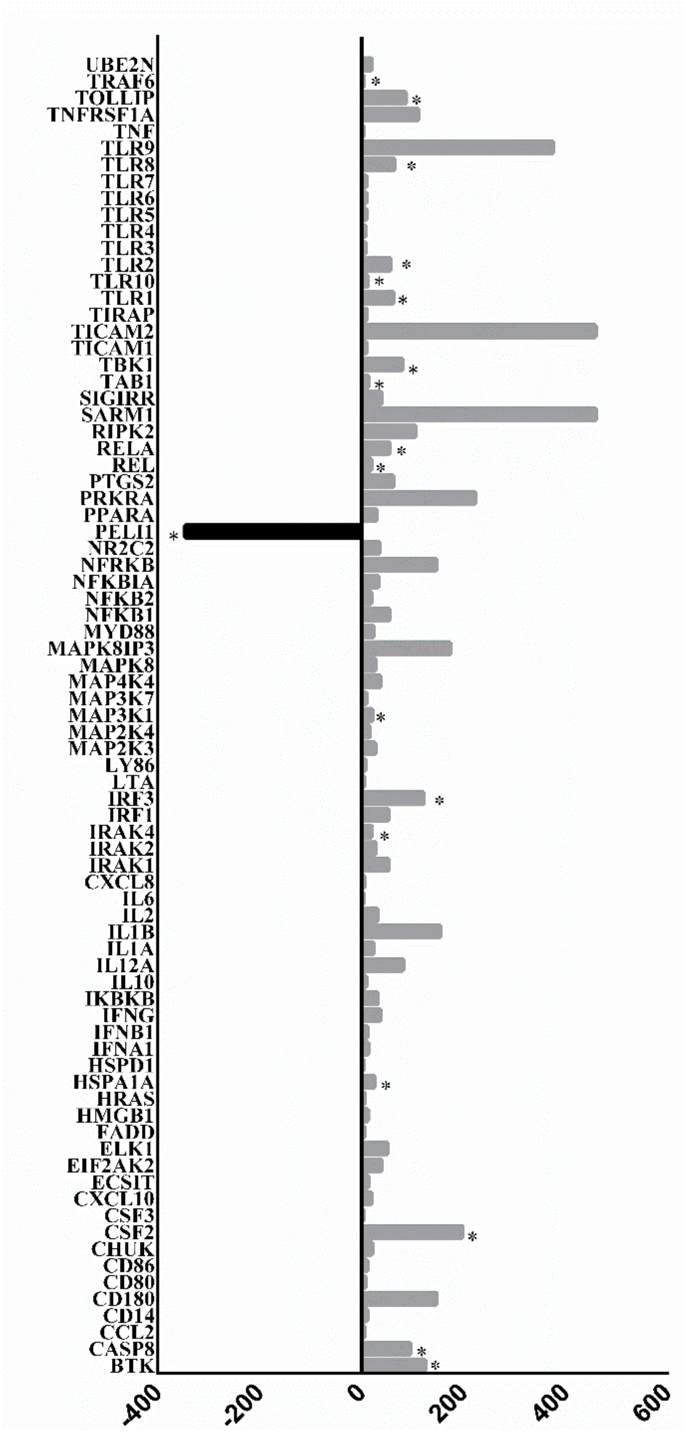

Figure S4. Gene expression between the moderate/severe vs asymptomatic groups. Upregulation (gray) and downregulation (black) of genes linked to immune response activation pathways. Statistical significance ($p < 0.05$) represented by an asterisk (*) (nonparametric Mann-Whitney-One-tailed test, GraphPad Prism® version 6.01).

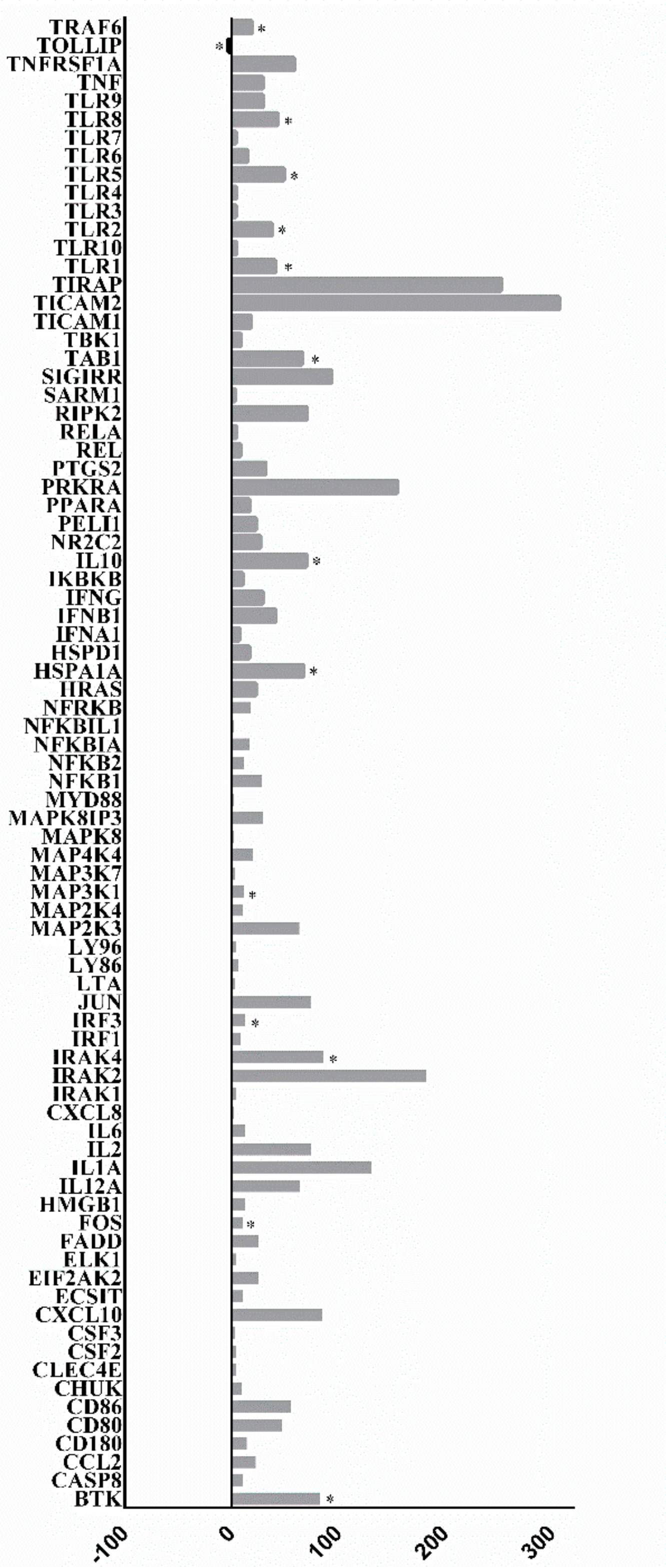

Figure S5. Gene expression between the moderate/severe vs mild groups. Upregulation (gray) and downregulation (black) of genes linked to immune response activation pathways. Statistical significance ($p < 0.05$) represented by an asterisk (*) (nonparametric Mann-Whitney-One-tailed test, GraphPad Prism® version 6.01).

Availability of data and materials

The DNA sequences generated and/or analysed during the current study are available in the GISAID EpiCoV repository (https://www.gisaid.org/) , Accession: EPI_ISL_13838063, EPI_ISL_13838064, EPI_ISL_13838065, EPI_ISL_13838066, EPI_ISL_13838067, EPI_ISL_13838024, EPI_ISL_13838068, EPI_ISL_13838025, EPI_ISL_13838069, EPI_ISL_13838026, EPI_ISL_13838027, EPI_ISL_13838028, EPI_ISL_13838029, EPI_ISL_13838060, EPI_ISL_13838061, EPI_ISL_13838062, EPI_ISL_13838052, EPI_ISL_13838053, EPI_ISL_13838054, EPI_ISL_13838055, EPI_ISL_13838056, EPI_ISL_13838057, EPI_ISL_13838058, EPI_ISL_13838059, EPI_ISL_13838090, EPI_ISL_13838091, EPI_ISL_13838092, EPI_ISL_13838093, EPI_ISL_13838050, EPI_ISL_13838094, EPI_ISL_13838051, EPI_ISL_13838041, EPI_ISL_13838085, EPI_ISL_13838042, EPI_ISL_13838086, EPI_ISL_13838043, EPI_ISL_13838087, EPI_ISL_13838044, EPI_ISL_13838088, EPI_ISL_13838045, EPI_ISL_13838089, EPI_ISL_13838046, EPI_ISL_13838047, EPI_ISL_13838048, EPI_ISL_13838049, EPI_ISL_13838080, EPI_ISL_13838081, EPI_ISL_13838082, EPI_ISL_13838083, EPI_ISL_13838040, EPI_ISL_13838084, EPI_ISL_13838030, EPI_ISL_13838074, EPI_ISL_13838031, EPI_ISL_13838075, EPI_ISL_13838032, EPI_ISL_13838076, EPI_ISL_13838033, EPI_ISL_13838077, EPI_ISL_13838034, EPI_ISL_13838078, EPI_ISL_13838035, EPI_ISL_13838079, EPI_ISL_13838036, EPI_ISL_13838037, EPI_ISL_13838038, EPI_ISL_13838039, EPI_ISL_13838070, EPI_ISL_13838071, EPI_ISL_13838072 and EPI_ISL_13838073). The others datasets generated and/or analyzed during the current study are available from the corresponding author on reasonable request.

List of abbreviations

ACTB: Actin, beta; AGA: Annotated Genome Aligner; B2M: Beta-2-microglobulin; BTK: Bruton agammaglobulinemia tyrosine kinase; CASP8: Caspase 8, apoptosis-related cysteine peptidase; CCL2: Chemokine (C-C motif) ligand 2; CD14: CD14 molecule; CD180: CD180 molecule; CD80: CD80 molecule; CD86: CD86 molecule; CDC: Centers for Disease Control and Prevention; CEPSH: Human Research Ethics Committee; CHUK: Conserved helix-loop-helix ubiquitous kinase; CLEC4E: C-type lectin domain family 4, member E; COPD: Chronic Obstructive Pulmonary Disease; COVID-19: Coronavirus Disease-2019; CSF2: Colony stimulating factor 2 (granulocyte-macrophage); CSF3: Colony stimulating factor 3 (granulocyte); Ct: Cycle Threshold; CXCL10: Chemokine (C-X-C motif) ligand 10; ECSIT: ECSIT homolog (Drosophila); EIF2AK2: Eukaryotic translation initiation factor 2-alpha kinase 2; ELK1: ELK1, member of ETS oncogene family; FADD: Fas (TNFRSF6)-associated via death domain; FOS: FBJ murine osteosarcoma viral oncogene homolog; GAPDH: Glyceraldehyde-3-phosphate dehydrogenase; GTR: General Time Reversible; HGDC: Human Genomic DNA Contamination; HMGB1: High mobility group box 1; HPRT1: Hypoxanthine phosphoribosyltransferase 1; HRAS: V-Ha-ras Harvey rat sarcoma viral oncogene homolog; HSPA1A: Heat shock 70kDa protein 1A; HSPD1: Heat shock 60kDa protein 1 (chaperonin); IBGE: Brazilian Institute of Geography and Statistics; ICU: Intensive Care Unit; IFNA1: Interferon, alpha 1; IFNB1: Interferon, beta 1, fibroblast; IFNG: Interferon, gamma; IKBKB: Inhibitor of kappa light polypeptide gene enhancer in B-cells, kinase beta; IL10: Interleukin 10; IL12A: Interleukin 12A (natural killer cell stimulatory factor 1, cytotoxic lymphocyte maturation factor 1, p35); IL1A:

Interleukin 1, alpha; IL1B: Interleukin 1, beta; IL2: Interleukin 2; IL6: Interleukin 6 (interferon, beta 2); IL8: Interleukin 8; IRAK1: Interleukin-1 receptor-associated kinase 1; IRAK2: Interleukin-1 receptor-associated kinase 2; IRAK4: Interleukin-1 receptor-associated kinase 4; IRF1: Interferon regulatory factor 1; IRF3: Interferon regulatory factor 3; JUN: Jun proto-oncogene; LACEN: Central Laboratory ; LTA: Lymphotoxin alpha (TNF superfamily, member 1); LY86: Lymphocyte antigen 86; LY96: Lymphocyte antigen 96; MAP2K3: Mitogen-activated protein kinase kinase 3; MAP2K4: Mitogen-activated protein kinase kinase 4; MAP3K1: Mitogen-activated protein kinase kinase kinase 1; MAP3K7: Mitogen-activated protein kinase kinase kinase 7; MAP4K4: Mitogen-activated protein kinase kinase kinase kinase 4; MAPK8: Mitogen-activated protein kinase 8; MAPK8IP3: Mitogen-activated protein kinase 8 interacting protein 3; ML: Maximum Likelihood; MYD88: Myeloid differentiation primary response gene (88); NFKB1: Nuclear factor of kappa light polypeptide gene enhancer in B-cells 1; NFKB2: Nuclear factor of kappa light polypeptide gene enhancer in B-cells 2 (p49/p100); NFKBIA: Nuclear factor of kappa light polypeptide gene enhancer in B-cells inhibitor, alpha; NFKBIL1: Nuclear factor of kappa light polypeptide gene enhancer in B-cells inhibitor like 1; NFRKB: Nuclear factor related to kappaB binding protein; NR2C2: Nuclear receptor subfamily 2, group C, member 2; PANGOLIN: Phylogenetic Assignment of Named Global Outbreak LINeages; PELI1: Pellino homolog 1 (Drosophila); PPARA: Peroxisome proliferator-activated receptor alpha; PPC: Positive PCR Control; PRKRA: Protein kinase, interferon-inducible double stranded RNA dependent activator; PTGS2: Prostaglandin-endoperoxide synthase 2 (prostaglandin G/H synthase and cyclooxygenase); REL: V-rel reticuloendotheliosis viral oncogene homolog (avian); RELA: V-rel reticuloendotheliosis viral oncogene homolog A (avian); RIPK2: Receptor-interacting serine-threonine kinase 2; RPLP0: Ribosomal protein, large, P0; RTC: Reverse

Transcription Control; RT-qPCR: Reverse Transcriptase Quantitative Polymerase Chain Reaction; SARM1: Sterile alpha and TIR motif containing 1; SARS-CoV-2: Severe Acute Respiratory Syndrome Coronavirus-2; SH-aLRT: Shimodaira–Hasegawa-like procedure; SIGIRR: Single immunoglobulin and toll-interleukin 1 receptor (TIR) domain; TAB1: TGF-beta activated kinase 1/MAP3K7 binding protein 1; TBK1: TANK-binding kinase 1; TICAM1: Toll-like receptor adaptor molecule 1; TICAM2: Toll-like receptor adaptor molecule 2; TIRAP: Toll-interleukin 1 receptor (TIR) domain containing adaptor protein; TLR1: Toll-like receptor 1; TLR10: Toll-like receptor 10; TLR2: Toll-like receptor 2; TLR3: Toll-like receptor 3; TLR4: Toll-like receptor 4; TLR5: Toll-like receptor 5; TLR6: Toll-like receptor 6; TLR7: Toll-like receptor 7; TLR8: Toll-like receptor 8; TLR9: Toll-like receptor 9; TNF: Tumor necrosis factor; TNFRSF1A: Tumor necrosis factor receptor superfamily, member 1A; TOLLIP: Toll interacting protein; TRAF6: TNF receptor-associated factor 6; UBE2N: Ubiquitin-conjugating enzyme E2N; WHO: World Health Organization;
